# Supplementary material for: Structural basis for arginine glycosylation of host substrates by bacterial effector proteins
Source: Nat Commun. 2018 Oct 16;9:4283. doi: 10.1038/s41467-018-06680-6 (PMC6191443; doi:10.1038/s41467-018-06680-6)
Supplement: Supplementary file 1 — Supplementary Information [file 41467_2018_6680_MOESM1_ESM.pdf]

Supplementary Information

Structural basis for arginine glycosylation of host substrates by bacterial effector proteins

J. B. Park, Y. H. Kim et al.

a

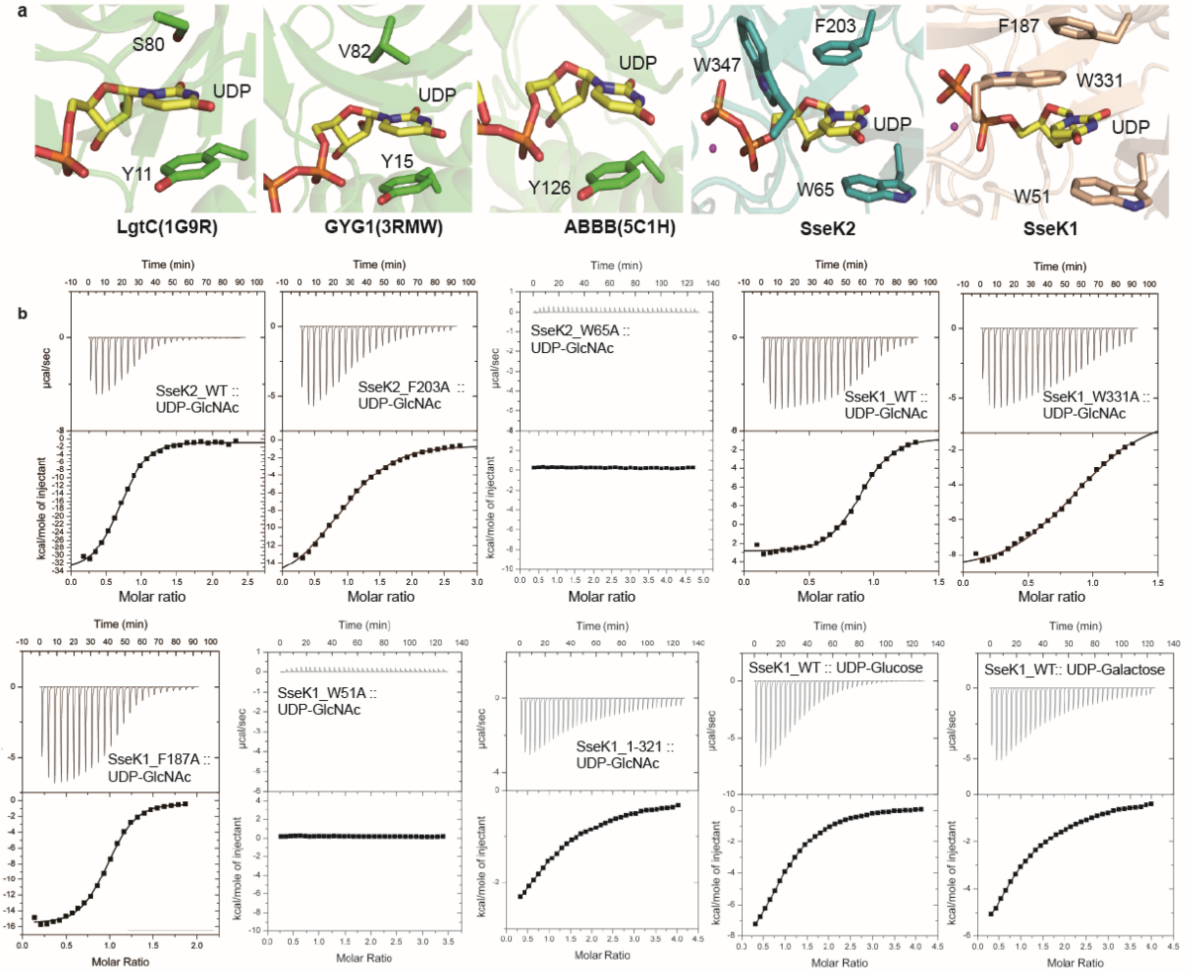

c

Ligand	UDP-GlcNAc					UDP-Glucose	UDP-Galactose
Protein	SseK1_WT	SseK1_W331A	SseK1_F187A	SseK1_W51A	SseK1_1-321	SseK1_WT	SseK1_WT
Chi2	4209	11521	17870	361	368	18669	1209
N	0.9	0.9	1	N.D	0.9	1	0.9
ΔH (Kcal/mol)	-13.4	-9.1	-15.9		-7	-9.2	-10.9
-TΔS (Kcal/mol)	4.8	2.3	7.1		1.9	2.6	4.8
ΔG (Kcal/mol)	-8.6	-6.8	-8.8		-5.1	-6.6	-6.1
Kd (uM)	2.3	23	2.9		357.1	37.9	159

Ligand	UDP-GlcNAc		
Protein	SseK2_WT	SseK2_F203A	SseK2_W65A
Chi2	8695	5763	5008
N	0.9	1	N.D
ΔH (Kcal/mol)	-15.4	-16.7	
-TΔS (Kcal/mol)	5.3	8.5	
ΔG (Kcal/mol)	-10.1	-8.2	
Kd (uM)	1.2	15.6	

d

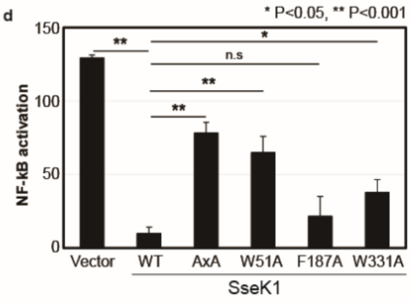

Supplementary Figure 1: The difference in the donor substrate binding mode between SseK1 and SseK2. a, SseK1 and SseK2 has either a Phe-Trp pair or a Trp-Trp pair for π-π

stacking interaction with the uridine of UDP-GlcNAc. Such an aromatic ring pair is an unusual structure among the GTs. The yellow stick shows a donor substrate and the four letters in the parentheses indicates the protein data bank (PDB) code number. **b**, Point mutations of each Phe-Trp pair or Trp-Trp pair and lid domain truncation form were constructed and an ITC assay was performed to determine the K_d value with UDP-GlcNAc. UDP-glucose and UDP-galactose affinity of SseK1 also measured by ITC assay. ITC data were prepared by using *Origin* (MicroCal, LLC) **c**, Summary of thermodynamic parameters from ITC experiments. Data integration, correction and analysis were carried out using *Origin* 7 ver. 7.0552 (MicroCal) with a single-site binding model. **d**, The NF-κB level in A549-NF-κB luc cells was measured to investigate enzymatic functions. Data represent at least three repetitions.

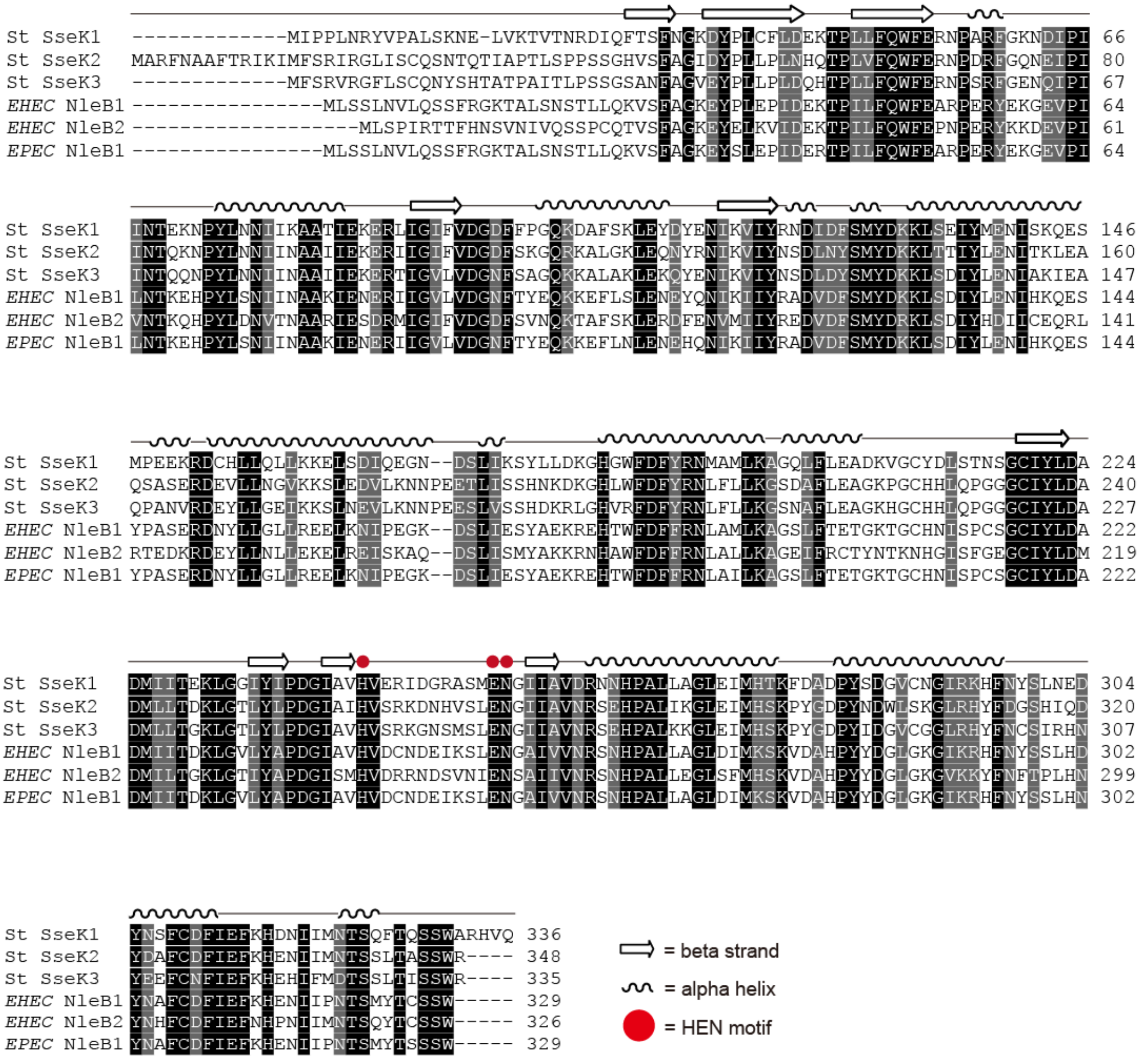

Supplementary Figure 2: Protein sequence alignment between SseK and NleB families. Perfectly conserved residues are highlighted in black and those that are similar among the sequences are highlighted in gray. The rest of the residues are shown in white. The figures were prepared using the Color Align Conservation in http://www.protocol-online.org/tools/sms2/color_align_cons.html website. *St= *Salmonella typhimurium SL1344*, EHEC = Enterohemorrhagic *Escherichia coli*, EPEC = Enteropathogenic *Escherichia coli*.

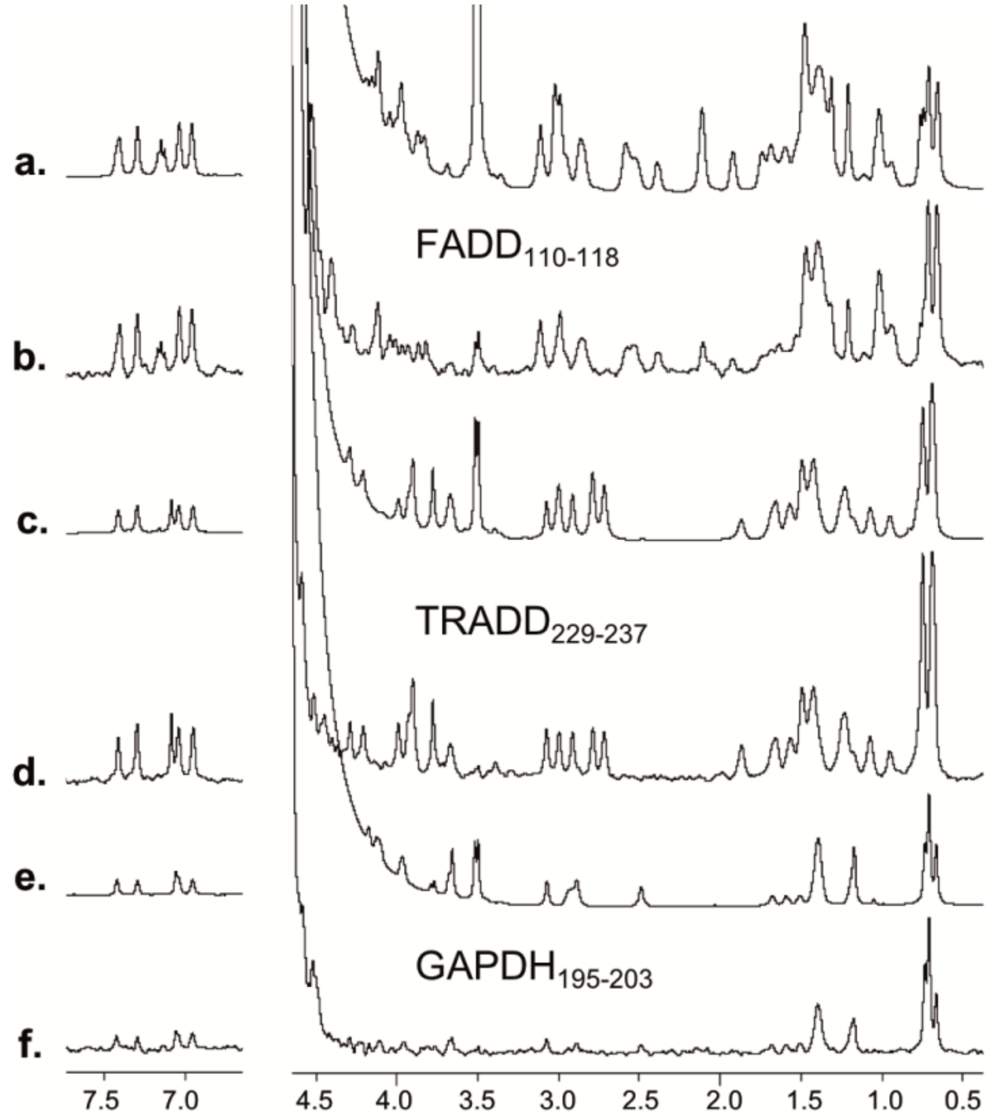

Supplementary Figure 3: Binding of short FADD, TRADD and GAPDH peptides to SseK1. Reference and STD NMR difference spectra of peptides in the presence of 25 μM SseK1, 25 μM $MnSO_4$, 25 μM UDP, and 25 mM Tris-d_{11} pH 7.4 in D_2O at 288 K (800 MHz). STD NMR difference spectra magnified 20x. **a**, **b**, Reference and STD NMR difference spectra of 1 mM $FADD_{110-118}$ respectively. **c**, **d**, Reference and STD NMR difference spectra of 1 mM $TRADD_{229-237}$ respectively. **e**, **f**, Reference and STD NMR difference spectra of 1 mM $GAPDH_{195-203}$ respectively.

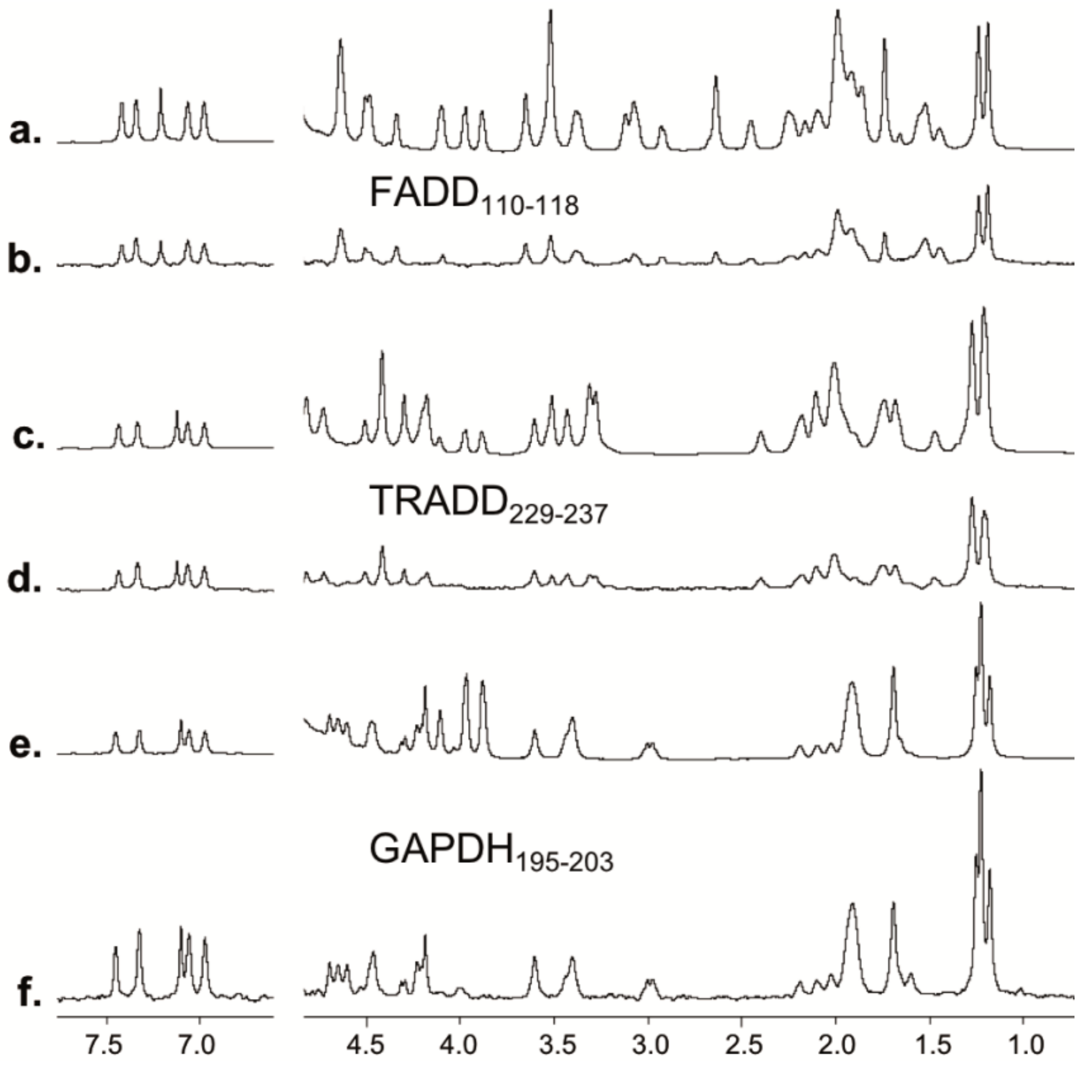

Supplementary Figure 4: Binding of short FADD, TRADD and GAPDH peptides to SseK2. Reference and STD NMR difference spectra of peptides in the presence of 25 μM SseK1, 25 μM $MnSO_4$, 25 μM UDP, and 25 mM Tris-d_{11} pH 7.4 in D_2O at 288 K (800 MHz). STD NMR difference spectra magnified 20x. **a**, **b**, Reference and STD NMR difference spectra of 1 mM $FADD_{110-118}$ respectively. **c**, **d**, Reference and STD NMR difference spectra of 1 mM $TRADD_{229-237}$ respectively. **e**, **f**, Reference and STD NMR difference spectra of 1 mM $GAPDH_{195-203}$ respectively.

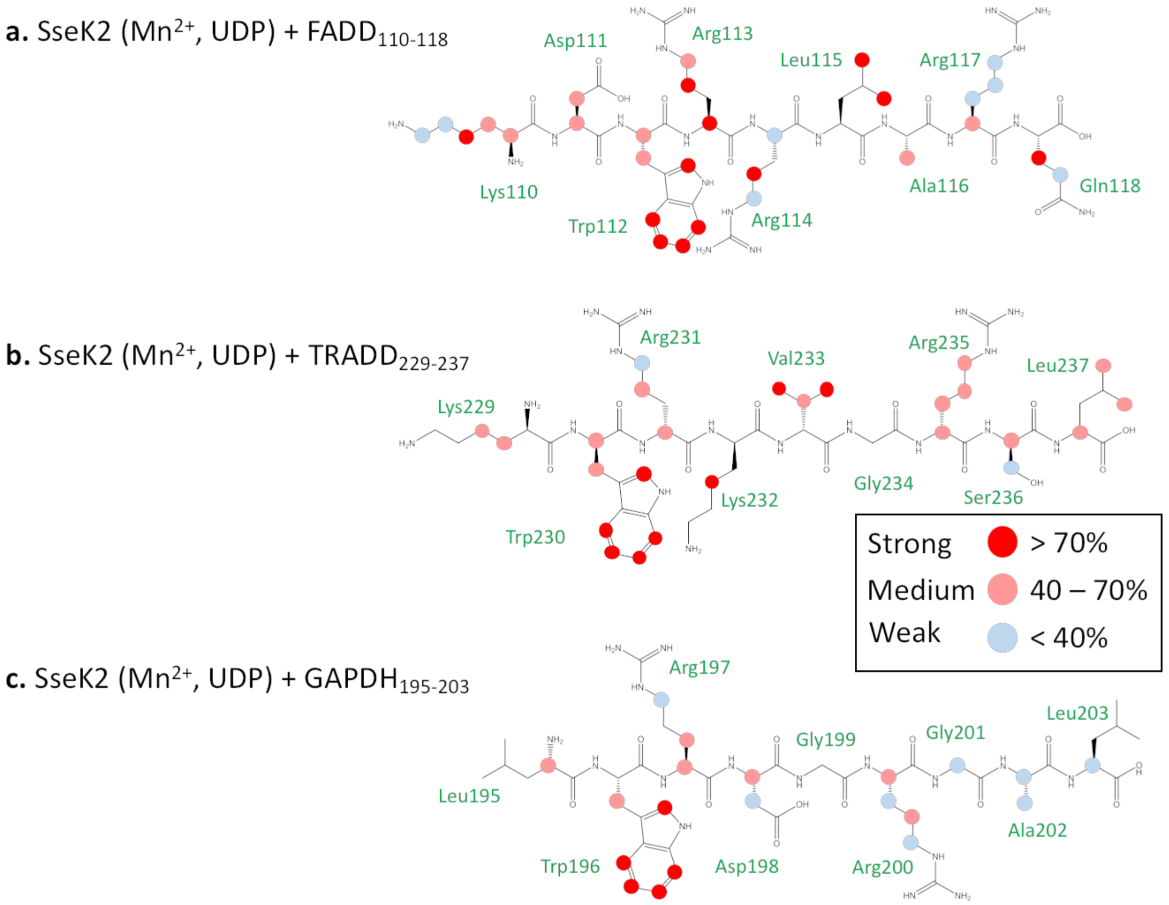

Supplementary Figure 5: Binding modes of short FADD, TRADD, and GAPDH substrate peptides to SseK2 from STD NMR. Binding epitope mappings of **a**, $FADD_{110\text{-}118}$ **b**, $TRADD_{229\text{-}237}$ and **c**, $GAPDH_{195\text{-}203}$ peptides in the presence of 25 µM SseK2. Samples contained 25 µM Mn^{2+}, and 25 µM UDP. All STD intensities were normalized against the Hζ2 of the tryptophan. Colored circles represent magnitude of normalized intensities (blue: < 40%, pink: 40-70%, red: >70%).

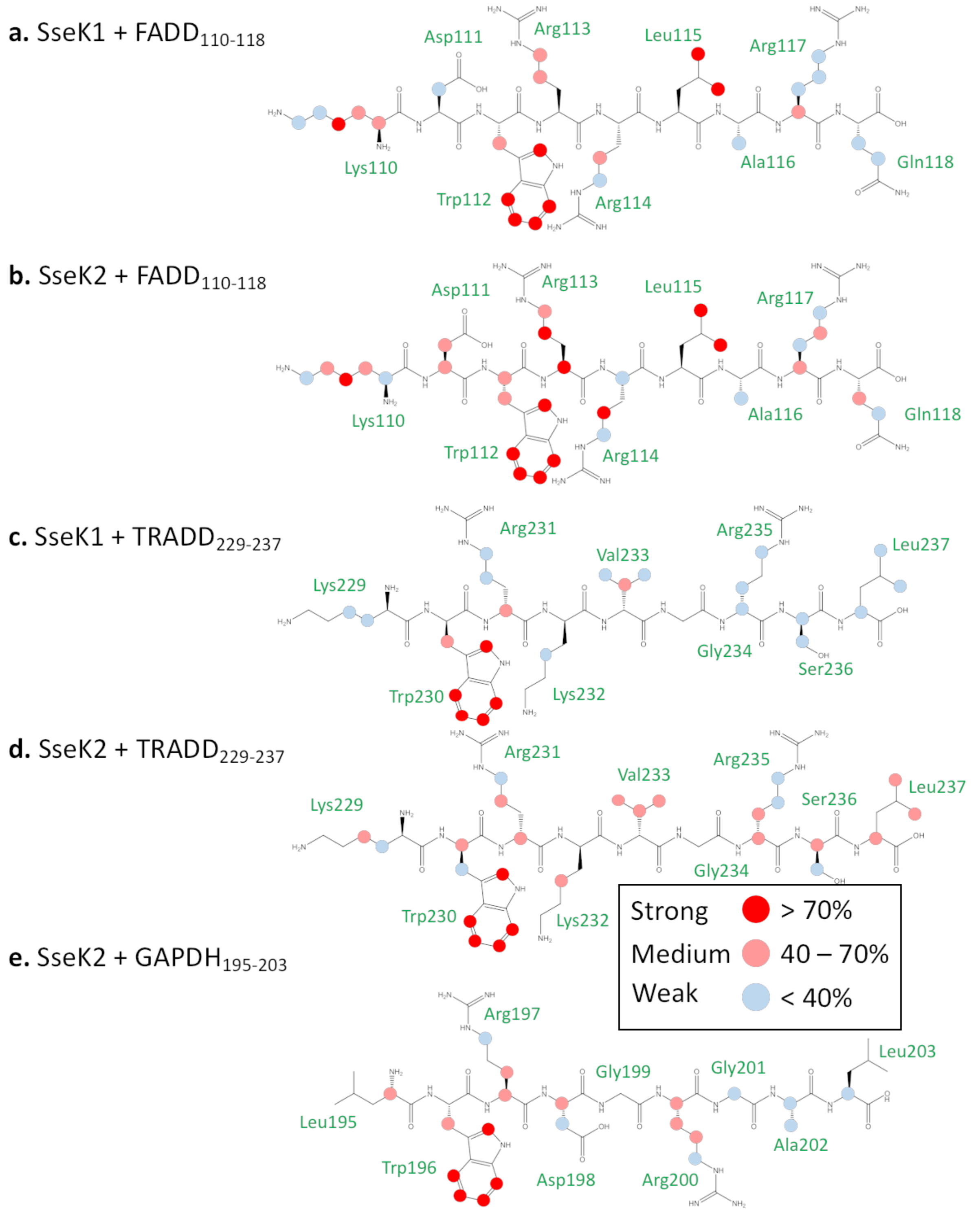

Supplementary Figure 6: Binding modes of short FADD, TRADD, and GAPDH substrate peptides to SseK1 and SseK2 in the absence of Mn^{2+} and UDP from STD NMR. Binding epitope mappings of **a**, $FADD_{110-118}$ binding to SseK1 **b**, $FADD_{110-118}$ binding to SseK2, **c**, $TRADD_{229-237}$ binding to SseK1, **d**, $TRADD_{229-237}$ binding to SseK2, **e**,

GAPDH$_{195\text{-}203}$ binding to SseK2. The binding epitope of GAPDH$_{195\text{-}203}$ binding to SseK1 in the absence of UDP is in **Fig. 5c** (main text). Samples contained 25 μM SseK1 or SseK2 and 25 μM Mn^{2+}. All STD intensities were normalized against the Hζ2 of the tryptophan. Colored circles represent magnitude of normalized intensities (blue: < 40%, pink: 40-70%, red: >70%). Except for GAPDH$_{195\text{-}203}$ binding to SseK1, all the peptides show similar binding modes in the presence or the absence of Mn^{2+} and UDP.

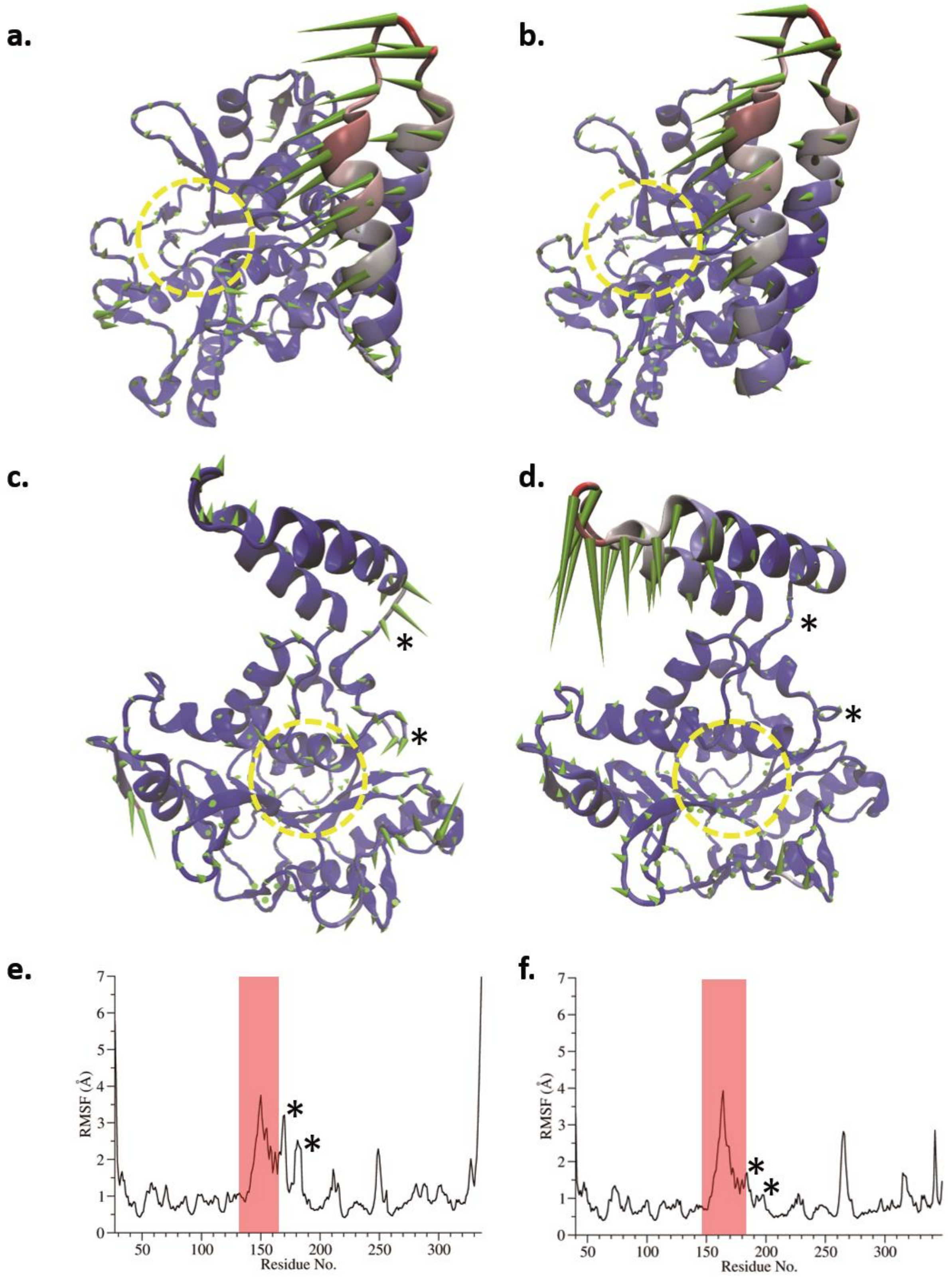

Supplementary Figure 7: Long Gaussian accelerated MD simulations predict significant differences in the dynamics of HLH domain between SseK1 and SseK2. **a**, **b**, Cartoon

representation of SseK1 (left) and SseK2 (right), showing the first eigenvector from principal component analysis. Green cones indicate the direction of motion. The magnitude of motion is represented both by cone length and by cartoon color scheme [high (red) – low (blue)]. The UDP-binding site is highlighted by a dashed yellow circle. **c**, **d**, Cartoon representation of SseK1 (left) and SseK2 (right), showing the second eigenvector from principal component analysis. Green cones indicate direction of motion. Magnitude of motion represented both by cone length and by cartoon color scheme [high (red) – low (blue)]. UDP-binding site highlighted by dashed yellow circle. Asterisks show regions with significant differences in motional fluctuations between SseK1 and SseK2, as determined from backbone RMSF. **e**, **f**, Backbone RMSF for each residue of SseK1 (left) and SseK2 (right). Red box shows HLH region. Asterisks highlight regions with significant differences between SseK1 and SseK2.

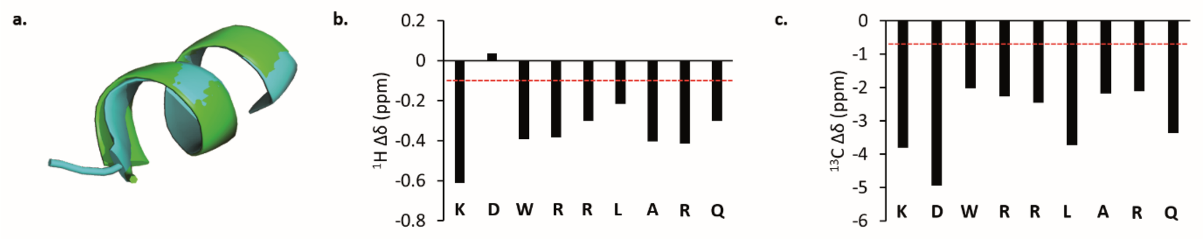

Supplementary Figure 8: FADD$_{110-118}$ remains in solution in its native helical conformation, as predicted from NMR chemical shift indexing. a, Overlaid structures of FADD$_{110-118}$ as predicted by PEPFOLD3[1] (green) and the equivalent region from the crystal structure of FADD (cyan; PDB 3EZQ). **b**, **c**, Difference between random coil and observed **b**, ^{1}H and **c**, ^{13}C chemical shifts for FADD$_{110-118}$. Random coil shifts are corrected for neighbors[2], temperature and pH[3]. Threshold for helicity shown as red dotted line[4,5].

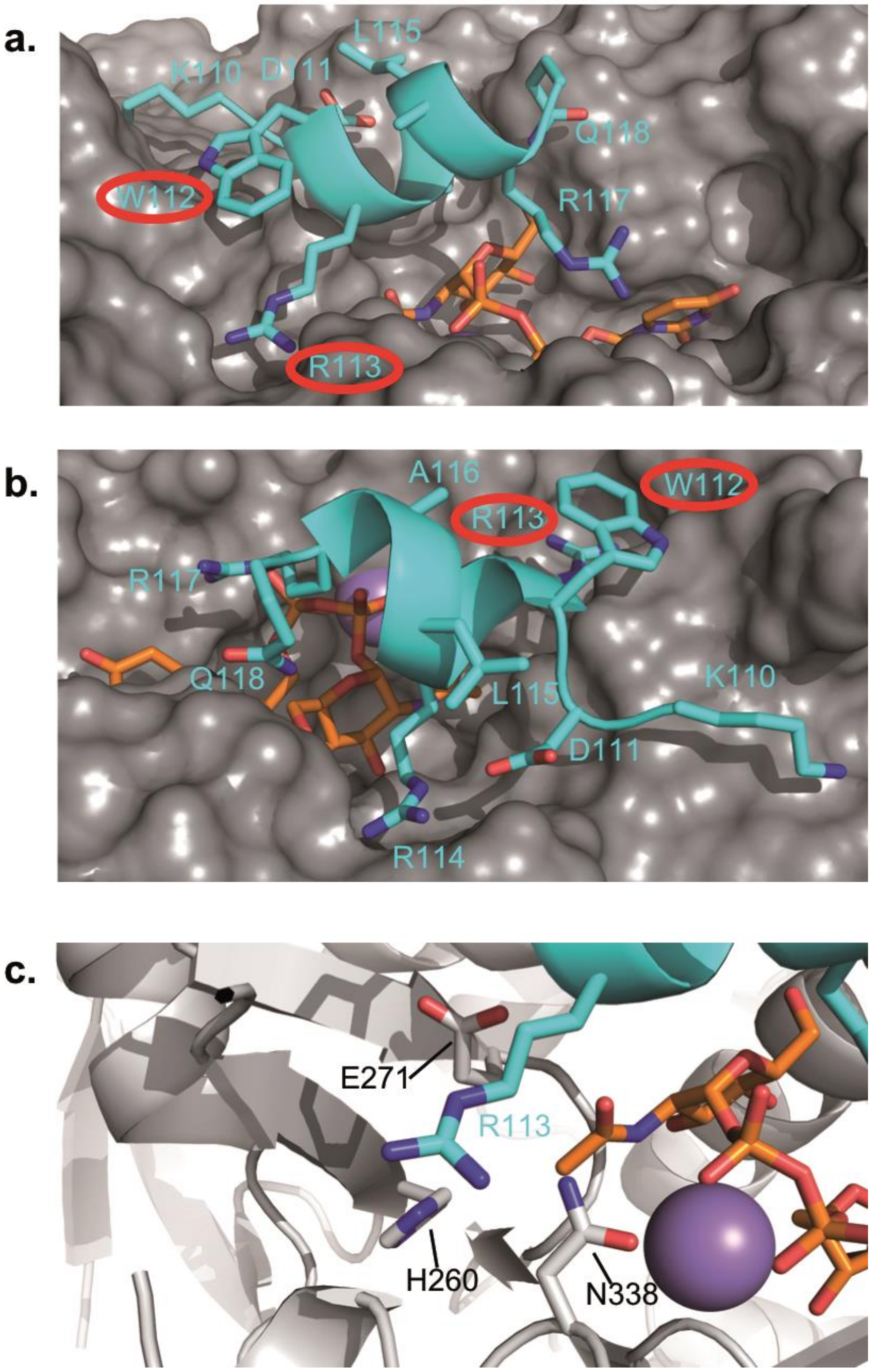

Supplementary Figure 9: Induced fit docking 3D molecular model, in agreement with STD NMR data, of the complex of SseK2 with UDP-GlcNAc and FADD$_{110\text{-}118}$. a, **b**, **c**, Different views of the 3D molecular model of the complex between SseK2 (grey surface) and FADD$_{110\text{-}118}$ (blue cartoon/sticks). UDP-GlcNAc shown as orange sticks. In **a**, and **b**, red circles indicate the WR motif that shows strongest STD signals. Hydrogen atoms omitted for clarity.

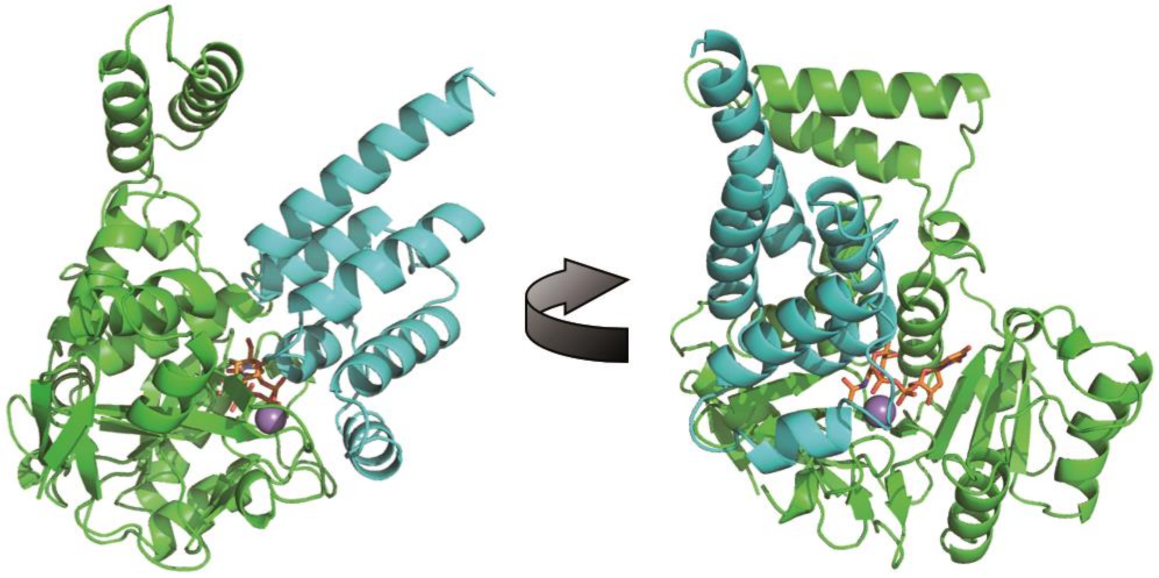

Supplementary Figure 10: 3D molecular model of the complex of SseK2 with UDP-GlcNAc and full FADD. Cartoon representation of the 3D model of the SseK2:UDP-GlcNAc:FADD complex generated by grafting the FADD crystal structure (3EZQ) onto the previously docked $FADD_{110\text{-}118}$ structure. SseK2 shown in green, FADD in blue, Mn^{2+} as a purple sphere, and UDP-GlcNAc as orange sticks. Hydrogen atoms omitted for clarity.

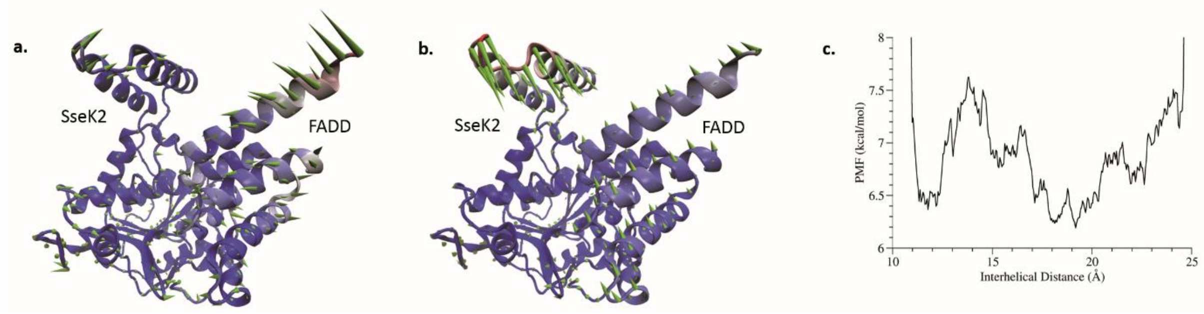

Supplementary Figure 11: Molecular dynamics of the 3D molecular model of the complex of SseK2 with UDP-GlcNAc and full FADD. Cartoon representation of the SseK2:UDP-GlcNAc:FADD complex, showing **a**, the first and **b**, second eigenvectors from principal component analysis. Green cones indicate direction of motion. Magnitude of motion represented both by cone length and by carton color scheme [high (red) – low (blue)]. **c**, Potential of mean force calculated as a function of interhelical distance by reweighting boost potentials from Gaussian aMD simulations. Reweighting was calculated by cumulant expansion to the second order. Interhelical distance defined as distance between the center of mass of the backbone heavy atoms of SseK2$_{168\text{-}177}$ and FADD$_{170\text{-}177}$.

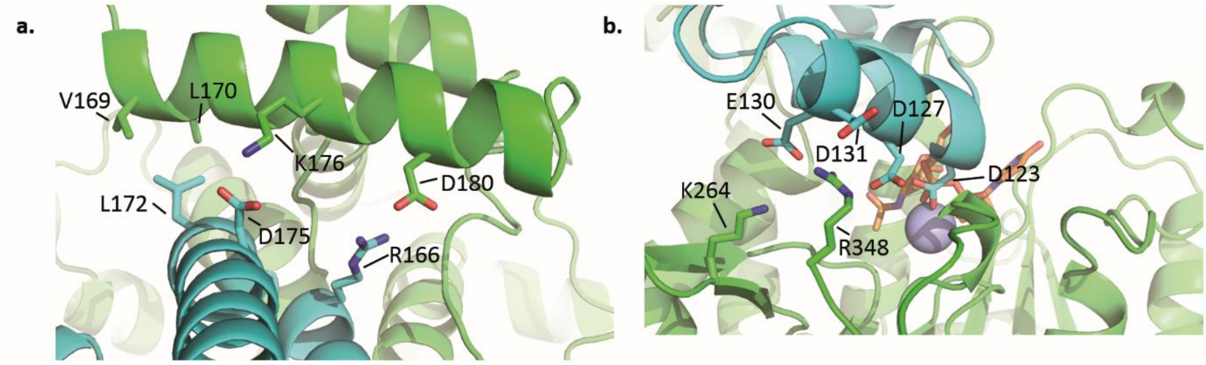

Supplementary Figure 12: Molecular dynamics of the 3D molecular model of the complex of SseK2 with UDP-GlcNAc and FADD. **a** and **b**, Representative structure of the SseK2:UDP-GlcNAc:FADD complex from GaMD simulations, showing close helical contact between the two proteins. Cartoon representation of SseK2 (cyan) and FADD (green) backbone, key sidechains shown as sticks, UDP as orange sticks, and Mn^{2+} as purple sphere. Hydrogen atoms are omitted for clarity.

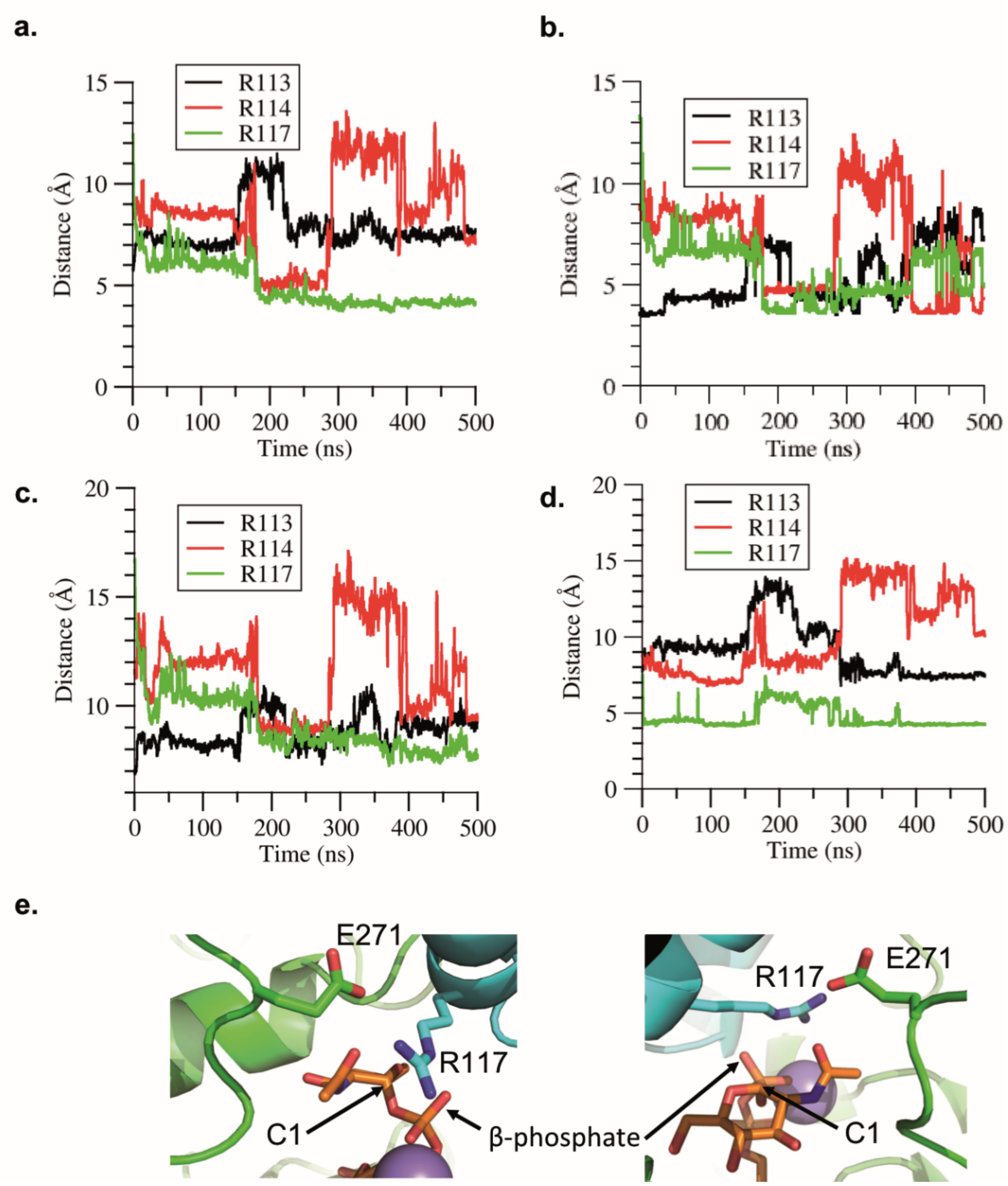

Supplementary Figure 13: Molecular dynamics of the docking model of the ternary SseK2:UDP-GlcNAc:FADD complex show significant conformational rearrangements of Arg113, Arg114, and Arg117 of FADD, orienting Arg117 for a front face attack to GlcNAc, and support the relevance of Glu271 and His260 in acceptor FADD substrate binding. Representative distances (Å) for contacts between the center of mass of the guanidinium groups of Arg113 (black lines), Arg114 (red lines), and Arg117 (green lines) of FADD and **a**, the anomeric carbon of GlcNAc of UDP-GlcNAc, **b**, the center of mass of the carboxylate group of Glu271 of SseK2, **c**, the center of mass of the imidazole group of His260 of SseK2, **d**, the center of mass of the beta-phosphate of UDP-GlcNAc. On average,

Arg117 of FADD is the residue from FADD closest to the anomeric carbon of GlcNAc and is the only residue establishing close contacts with His260, Glu271, and the beta-phosphate of UDP-GlcNAc. **e**, Representative structure of the ternary complex of SseK2 (green), FADD (cyan) and UDP-GlcNAc (orange) from GaMD simulations, showing that Arg117 is properly oriented for a front face attack with close contact with the anomeric carbon of the UDP-GlcNAc donor substrate. Manganese is shown as a purple sphere and hydrogen atoms are omitted for clarity.

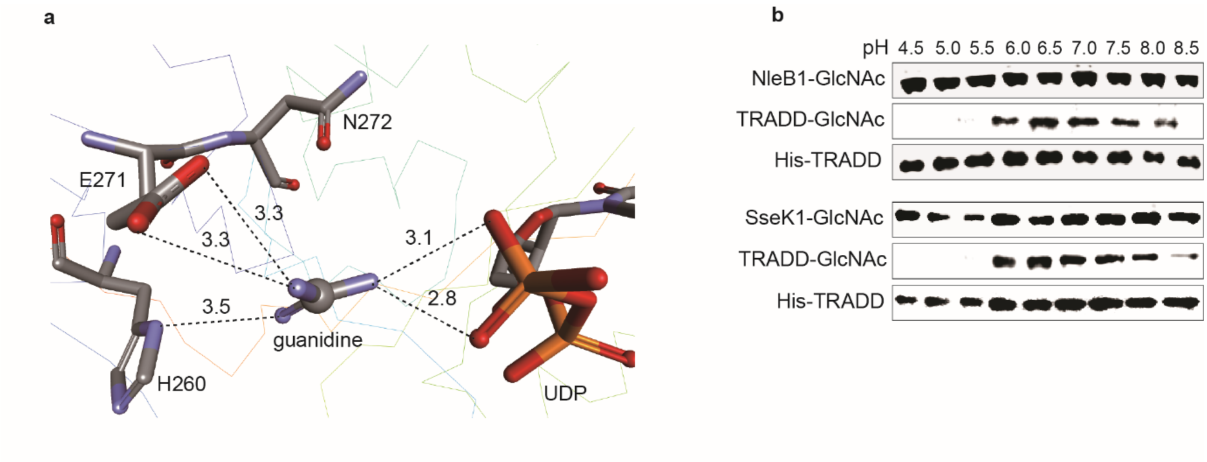

Supplementary Figure 14: Docking structure of acceptor substrate binding mode. a, Guanidine docking at the active site of the UDP bound SseK2 crystal structure. His260, Glu271, and the β-phosphate of UDP interact with guanidine via hydrogen bonding. Dashed lines represent hydrogen bonds. **b**, pH-dependency of NleB1 and SseK1. Glycosylated TRADD was detected using a GlcNAc-arginine specific antibody.

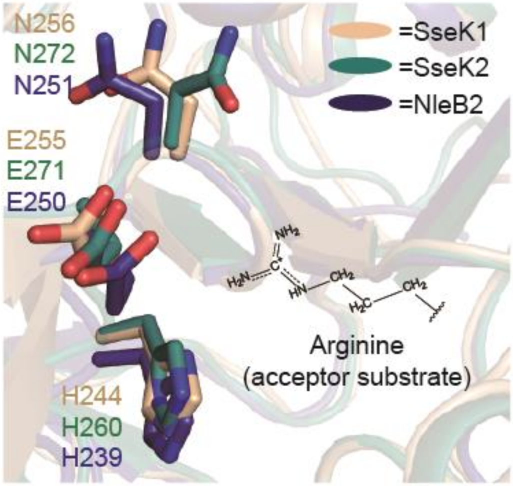

Supplementary Figure 15: Structural conservation of the HEN motif. Superimposition of SseK1, SseK2, and NleB2 based on the C-α and each HEN motif is shown in stick shape. Acceptor substrate (arginine) was sketched in the putative binding site based on the docking model. The three structures are expressed in different colors.

a

SseK1	Km (uM-1)<br>*P-Value	Kcat (s-1)<br>*P-Value	Kcat/Km (uM-1/s-1)	Relative catalytic efficacies (%)
WT	0.06 ± 0.01<br>*0.0629	23466 ± 666.7<br>*<0.0001	379279.10	
DxD	0.19 ± 0.06<br>0.0019	4551 ± 230.67<br>*<0.0001	23398.46	6.17
H244A	0.27 ± 0.05<br>0.0216	7104 ± 221.27<br>*<0.0001	26676.68	7.03
E255A	0.13 ± 0.004<br>0.0379	16416 ± 188.8<br>*<0.0001	124741.60	32.89
N256A	0.48 ± 0.06<br>0.0049	4148 ± 168.88<br>*<0.0001	8674.20	2.29

SseK2	Km (uM-1)<br>*P-Value	Kcat (s-1)<br>*P-Value	Kcat/Km (uM-1/s-1)	Relative catalytic efficacies (%)
WT	0.33 ± 0.07<br>*0.0072	2026 ± 57.71<br>*0.0002	6093.23	
DxD	1.76 ± 0.56<br>*<0.0001	713.9 ± 69.60<br>*0.0044	405.63	6.66
H260A	1.75 ± 0.27<br>*0.0002	219.5 ± 48.28<br>*0.0561	125.64	2.06
E271A	0.91 ± 0.16<br>*0.0006	494.2 ± 64.28<br>*0.0404	544.57	8.94
N272A	1.69 ± 0.28<br>*0.0003	400.4 ± 88.79<br>*0.1146	237.63	3.90

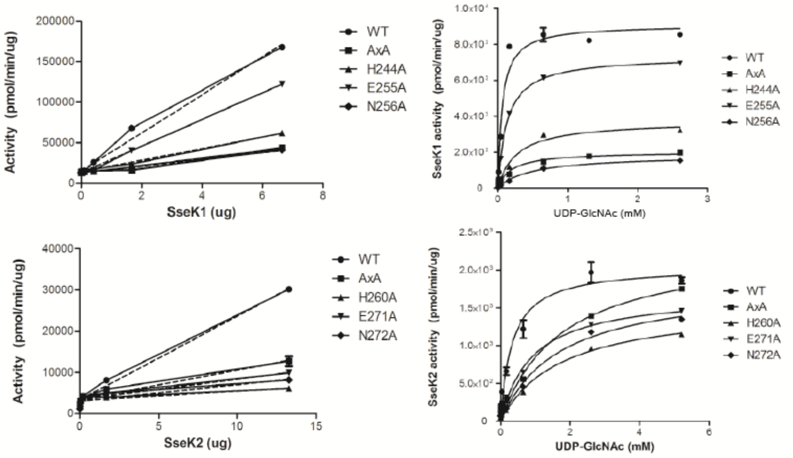

b

SseK1				
GAPDH	Km (uM)<br>*P-Value	Kcat (s-1)<br>*P-Value	Kcat/Km (uM/s-1)	Relative catalytic efficacies (%)
WT	2.36 ± 0.74<br>*0.1273	101.00 ± 3.51<br>*<0.0001	42.83	
WA	3.19 ± 0.53<br>*0.0589	55.25 ± 3.74<br>*0.0001	17.34	40.49
RA	2.88 ± 0.73<br>*0.0913	58.44 ± 2.43<br>*0.0007	20.28	47.35
AA	4.97 ± 0.72<br>*0.0144	36.75 ± 2.27<br>*0.0005	7.40	17.28

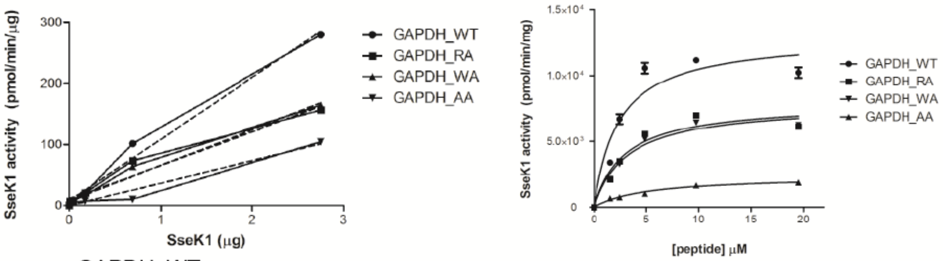

GAPDH_WT: TVDGPSGKLWRDGRGAL
GAPDH_WA (W196A): TVDGPSGKLARDGRGAL
GAPDH_RA (R197A): TVDGPSGKLWADGRGAL
GAPDH_AA (W196A, R197A): TVDGPSGKLAADGRGAL

Supplementary Figure 16: Enzyme kinetics assay using recombinant proteins. **a**, Point mutant constructs were generated by PCR based point-mutagenesis and purified by Ni-NTA and size-exclusion chromatography. UDP-Glo™ Glycosyltransferase Assay kit (Promega, #V6961) was carried out as per manufacturer's instructions. L-arginine (> 98.5% purity, Duchefa BIOCHEMIE) was used as an acceptor substrate. **b**, SseK1 enzyme kinetics assay data for $GAPDH_{187-203}$ synthetic peptide and mutant forms of the WR motif. Plates were read using a VICTOR5 (PerkinElmer) device. Graph generation and calculation of each K_m and K_{cat} values are automatically calculated using the *GraphPad Prism* 5 software program. The data represent at least two repetitions. The final specific activity of the transfer reaction was corrected considering the hydrolysis reaction, which was performed using SseK1/SseK2, UDP-GlcNAc and $MnCl_2$. Plus-minus values are mean ± SE.

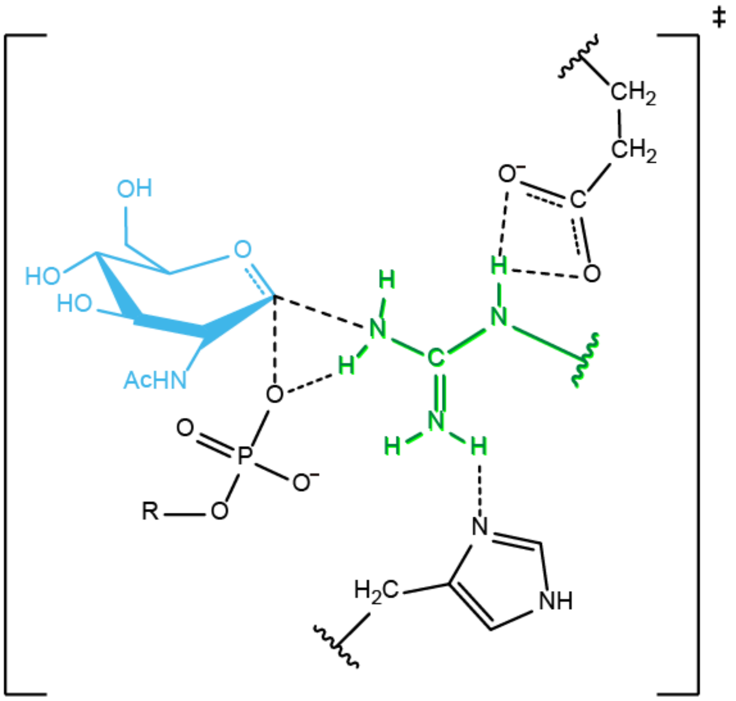

Supplementary Figure 17: Proposed SseK mechanism. See text for details.

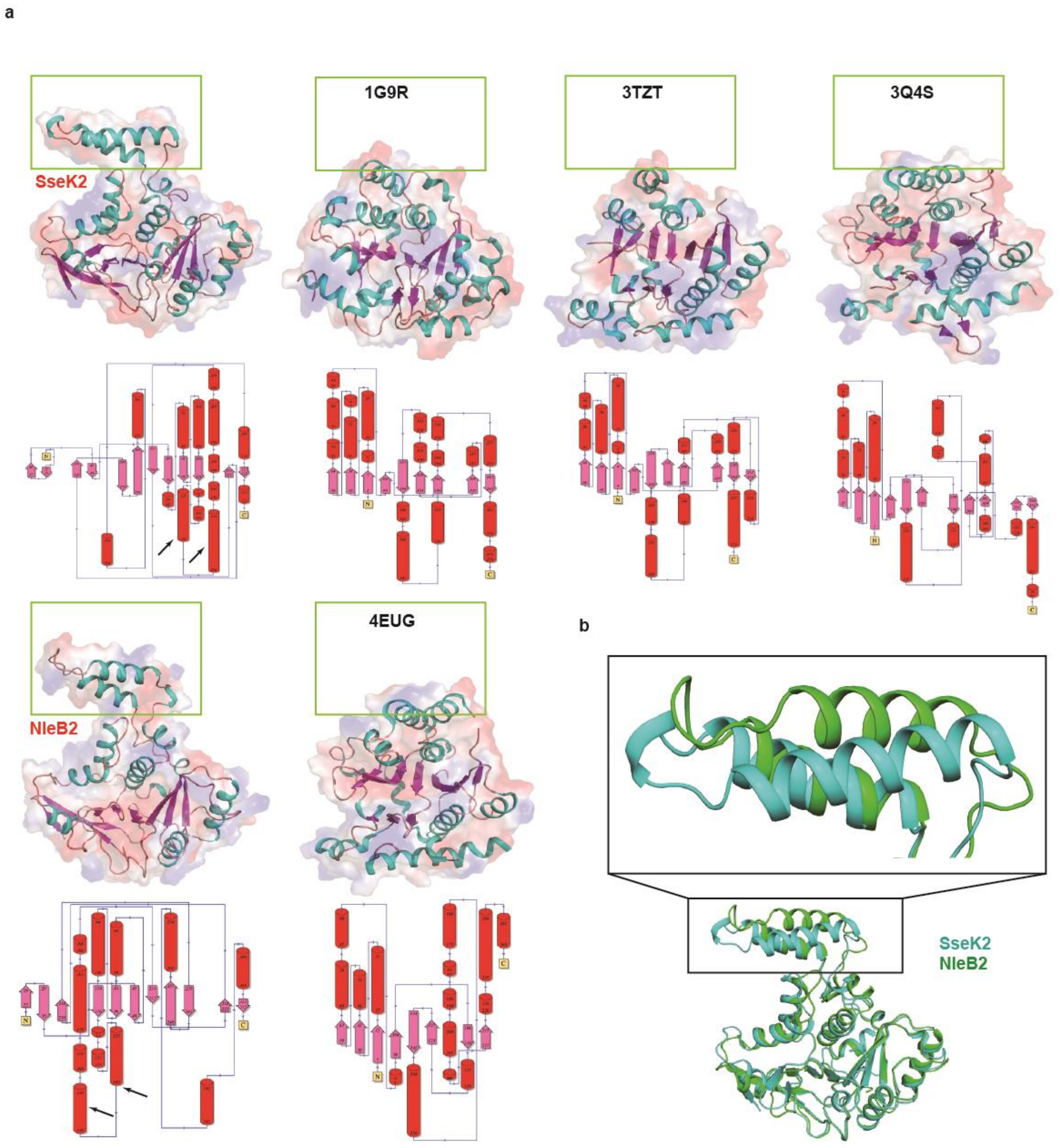

Supplementary Figure 18: Flexible structure of the HLH domain. SseK and NleB have a protruding helix-loop-helix (HLH) domain. **a**, Protein structures were arranged based on beta-sheet (purple color) similarity and the HLH region is shown as a yellowish green quadrangle (each upper panel). Protein structure topology is also shown in the lower panel using the *PDBSUM* website. The HLH domains are indicated by black arrows. The four letters in the yellow-green rectangles indicate the PDB access codes. **b**, Superimposition between SseK1, SseK2, and NleB2 based on the amino acid backbone C-α carbons.

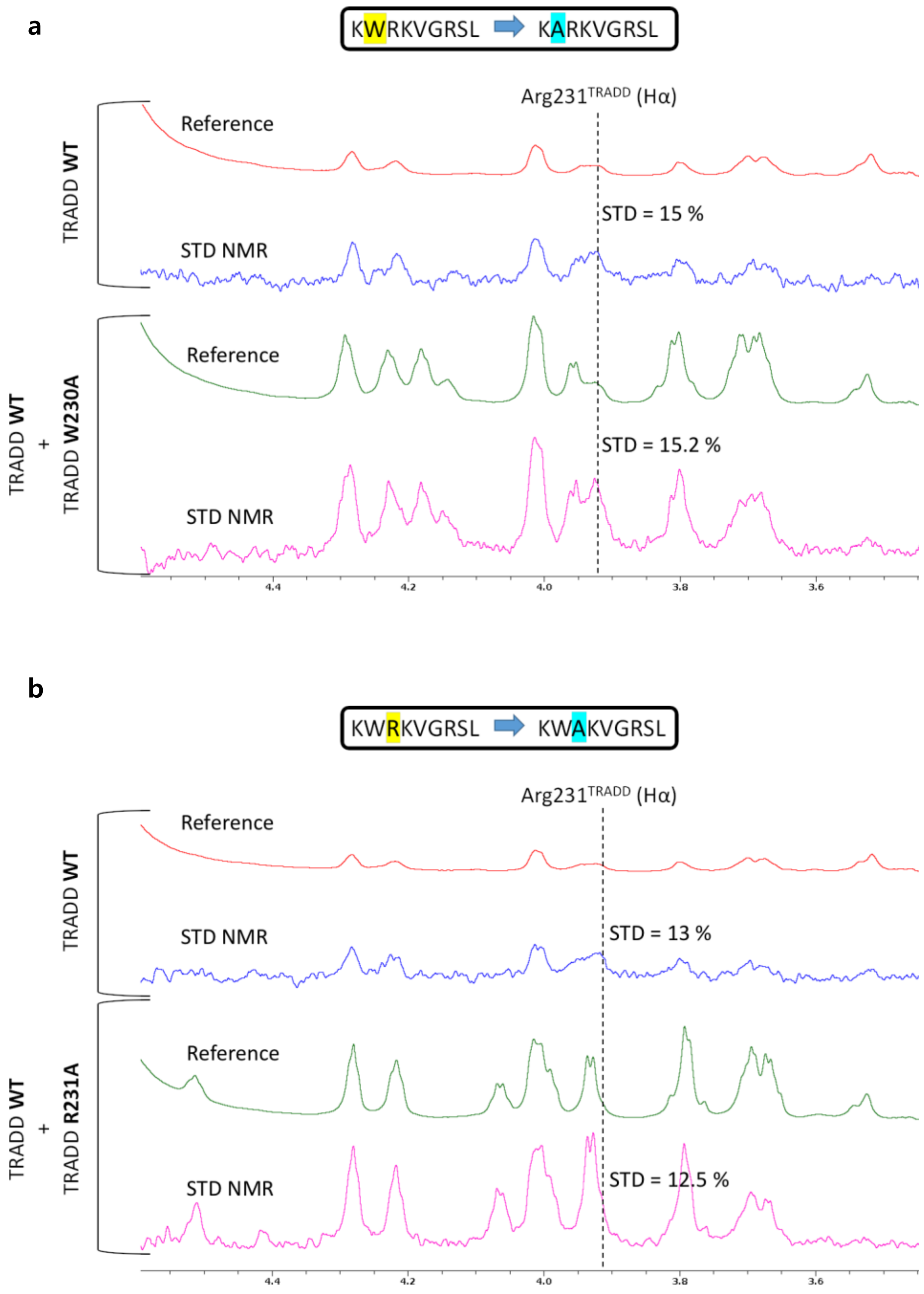

Supplementary Figure 19: STD NMR competition experiments of TRADD$_{229\text{-}237}$ wild-type and mutants. a, Competition of TRADD WT with single mutant TRADD W230A. Top: reference and STD NMR spectrum of TRADD WT in the presence of SseK1; Bottom: reference and STD NMR spectrum of the same sample after addition of an equimolar concentration of the mutant TRADD W230A. **b,** Competition of TRADD WT with single mutant TRADD R231A. Top: reference and STD NMR spectrum of TRADD WT in the presence of SseK1; Bottom: reference and STD NMR spectrum of the same sample after addition of an equimolar concentration of the mutant TRADD R231A. Within the experimental error, the STD intensities of TRADD WT are not affected by the addition of the

mutants. This means that the affinity of the WT peptide is significantly higher than those of the mutants.

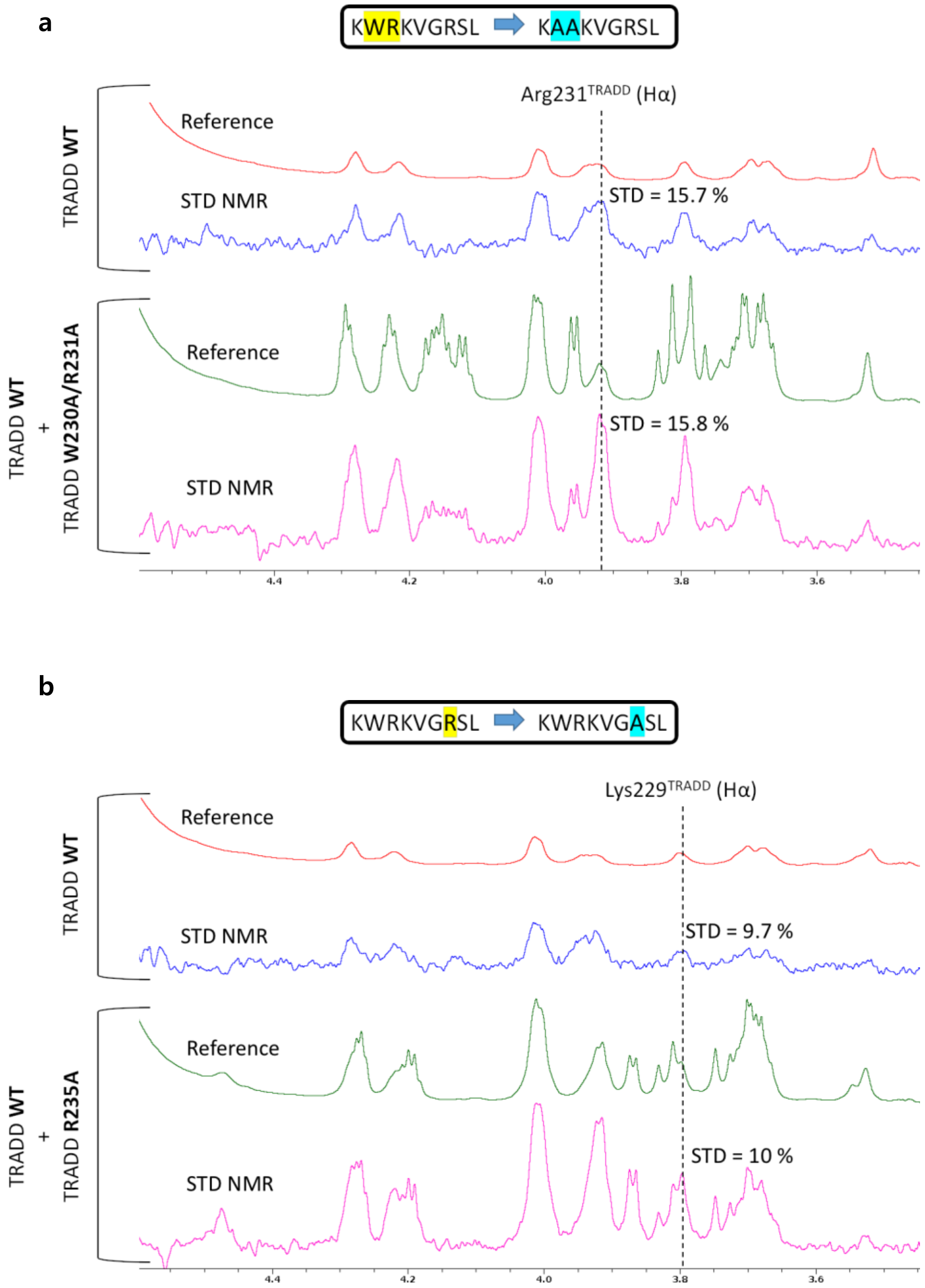

Supplementary Figure 20: STD NMR competition experiments of TRADD$_{229\text{-}237}$ wild-type and mutants. a, Competition of TRADD WT with double mutant TRADD W230A/R231A. Top: reference and STD NMR spectrum of TRADD WT in the presence of SseK1; Bottom: reference and STD NMR spectrum of the same sample after addition of an equimolar concentration of the mutant TRADD W230A. **b,** Competition of TRADD WT with single mutant TRADD R235A. Top: reference and STD NMR spectrum of TRADD WT in

the presence of SseK1; Bottom: reference and STD NMR spectrum of the same sample after addition of an equimolar concentration of the mutant TRADD R231A. Within the experimental error, the STD intensities of TRADD WT are not affected by the addition of the mutants. This means that the affinity of the WT peptide is significantly higher than those of the mutants.

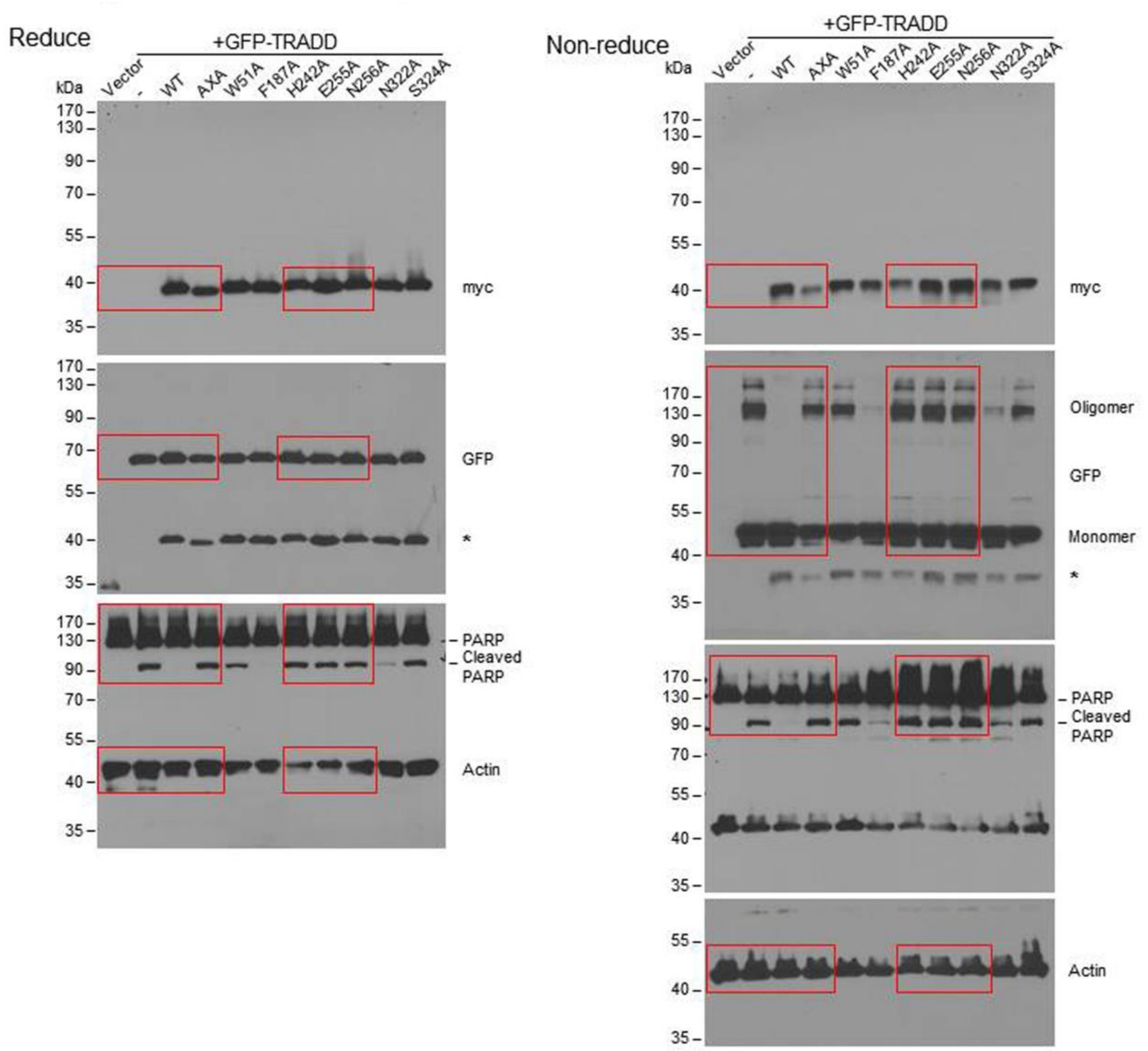

Supplementary Figure 21: Uncropped images of **Figure 6a** blots

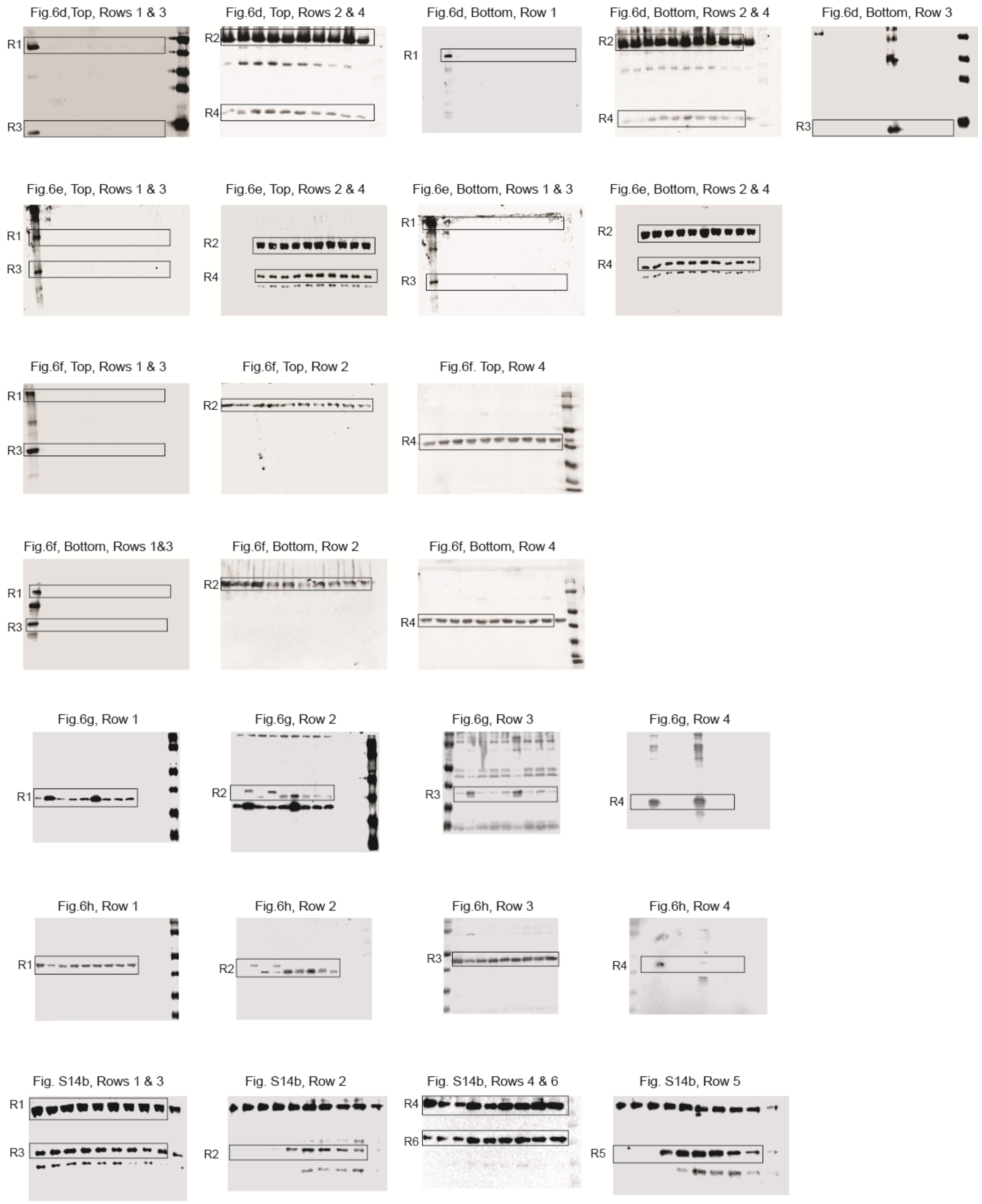

Supplementary Figure 22: Uncropped images of **Figure 6d-h** and **Supplementary Figure 14b** blots

Supplementary Table 1. Data collection and refinement statistics

	Seleno-Met NleB2 (SAD peak)	NleB2 **(5H5Y)**	SseK1_UDP **(5H60)**	SseK2 **(5H61)**	SseK2_UDP **(5H62)**	SseK2_UDP-GlcNAc **(5H63)**
**Data collection**						
Space group	$P2_12_12$	$P2_12_12$	$P6_222$	$P2_1$	$P2_1$	P1
Cell dimensions						
a, *b*, *c* (Å)	66.5, 95.9, 122.1	67.8, 95.5, 120.1	115.9, 115.9, 100.1	46.6, 145.6, 55.5	49.6, 143.6, 52.7	49.6, 52.3, 143.8
α, β, γ (°)	90, 90, 90	90, 90, 90	90, 90, 120	90, 105.1, 90	90, 108.2, 90	90, 90, 108
Resolution (Å)	60-3.0 (3.11-3.04)	67.8-2.1 (2.16-2.10)	70.9-3.15 (3.27-3.15)	72.8-1.9 (1.91-1.86)	47.3-1.7 (1.70-1.66)	47.9-1.9 (1.97-1.92)
R_{merge} (%)	9.2 (96)	11.1 (94.0)	14.9 (121.0)	5.6 (70)	6.4 (84)	7.6 (36.7)
$I / \sigma I$	17.4 (2.8)	15.5 (3.3)	20.6 (4.0)	15.8 (2.1)	16.3 (2.2)	9.2 (3.1)
Completeness (%)	100 (100)	99.7 (99.9)	99.9 (100)	98 (95.1)	99.5 (100)	94.4 (94.9)
Redundancy	6.3 (6.1)	13.6 (14.0)	28.1 (30.3)	5.5 (5.5)	7.0 (7.1)	2.6 (2.6)
Wilson B-factor ($Å^2$)	24.4	28.7	82.6	36.5	33.6	32.5
**Refinement**						
Resolution (Å)		44.4-2.1 (2.15-2.10)	70.9-3.15 (3.26-3.15)	53.6-1.9 (1.93-1.86)	41.4-1.7 (1.72-1.66)	41.1-1.9 (1.99-1.92)
No. reflections		45767 (2651)	6935 (354)	58396 (3943)	81824 (5786)	98965 (7027)
No. reflections used for R_{free}		43743	6927	55555	77783	94014
R_{work} / R_{free} (%)		24.3 (32.0) /29.1 (36.1)	27.4 (29.0) /35.7 (40.1)	20.2 (28.6) /23.6 (31.7)	19.0 (28.5) /21.8 (30.2)	18.4 (25.3) /22.8 (25.1)
No. atoms		4902	2499	5030	5516	10767
Protein		4662	2473	4765	4894	9854
Ligand/ion		-	26	-	65	162
Water		240	0	265	557	751
B-factors		45.1	89.1	37.0	29.7	28.0
Protein		45.3	92.7	35.4	28.7	28.7
Ligand/ion		-	85.5	-	24.2	21.0
Water		45.0	-	40.1	39.2	35.0

R.m.s. deviations						
Bond lengths (Å)		0.0082	0.0102	0.0211	0.0236	0.0203
Bond angles (°)		0.96	1.50	1.72	1.87	2.16
Ramachandran favored (%)		95.1	91.0	98.8	97.8	97.0
Ramachandran outliers (%)		0	0.7	0	0	0.16
Clashscore		9.0	36.0	13.1	7.26	6.46

*Values in parentheses are for highest resolution shell.

Supplementary Table 2. Primers used in this study

Description (Primers)	Sequence
**NleB1 XhoI F**	CGCGCTCGAGATGT$_2$ATCT$_2$CAT$_2$A$_3$TGTC$_2$T$_2$CA$_2$TC
**NleB1 KpnI F**	CGCG$_3$TAC$_4$ATGA$_2$CTGCAG$_2$TATACATACTG$_2$TAT$_2$
**NleB1 MseI F**	C$_2$AC$_2$ATGTAC$_3$ATACGATGT$_2$C$_2$AGAT$_2$ACGCTATGT$_2$ATCT$_2$CAT$_2$A$_3$TG
**NleB1 BamHI R**	CTCATCA$_2$TGTATCT$_2$ATCATGTCTG$_2$ATC$_4$T$_2$AC$_2$ATGA$_2$CTGCAG$_2$TATAC
**NleB1 H242A F**	CTC$_2$TGATG$_2$TATCGCTGTG$_2$CTGTAGAT$_2$GTA$_2$TG
**NleB1 H242A R**	CAT$_2$ACA$_2$TCTACAGC$_2$ACAGCGATAC$_2$ATCAG$_2$AG
**NleB1 E253A F**	GATGAGATA$_5$GTCT$_2$GCA$_3$TG$_2$TGCGATAGT$_2$G
**NleB1 E253A R**	CA$_2$CTATCGCAC$_2$AT$_3$GCA$_2$GACT$_5$ATCTCATC
**NleB1 N254A F**	GAGATA$_5$GTCT$_2$GA$_2$GCTG$_2$TGCGATAGT$_2$G
**NleB1 N254A R**	CA$_2$CTATCGCAC$_2$AGCT$_2$CA$_2$GACT$_5$ATCTC
**NleB1 H242A, E253A, N254A F**	GATG$_2$TATCGCTGTG$_2$CTGTAGAT$_2$GTA$_2$TGATGAGATA$_5$GTCT$_2$GCAGCTG$_2$TGCGATAG
**NleB1 H242A, E253A, N254A R**	CTATCGCAC$_2$AGCTGCA$_2$GACT$_5$ATCTCATCAT$_2$ACA$_2$TCTACAGC$_2$ACAGCGATAC$_2$ATC
**NleB2 H239A F**	GCTC$_2$TGATG$_2$A$_2$T$_3$CA$_2$TG$_2$CTGTG$_2$ATCGTCGTA$_2$TG
**NleB2 H239A R**	CAT$_2$ACGACGATC$_2$ACAGC$_2$AT$_2$GA$_3$T$_2$C$_2$ATCAG$_2$AGC
**NleB2 E250A F**	GTA$_2$TGATAGTGTA$_3$TAT$_2$GCTA$_2$TAGTGCA$_2$TA$_2$T$_2$GT$_2$A$_2$C
**NleB2 E250A R**	GT$_2$A$_2$CA$_2$T$_2$AT$_2$GCACTAT$_2$AGCA$_2$TAT$_3$ACACTATCAT$_2$AC
**NleB2 N251A F**	GTA$_2$TGATAGTGTA$_3$TAT$_2$GA$_2$GCTAGTGCA$_2$TA$_2$T$_2$GT$_2$A$_2$C
**NleB2 N251A R**	GT$_2$A$_2$CA$_2$T$_2$AT$_2$GCACTAGCT$_2$CA$_2$TAT$_3$ACACTATCAT$_2$AC
**NleB2 H239A, E250A, N251A F**	GATG$_2$A2T$_3$CA$_2$TG$_2$CTGTG$_2$ATCGTCGTA$_2$TGATAGTGTA$_3$TAT$_2$GCTGCTAGTGCA$_2$TA$_2$T$_2$G
**NleB2 H239A, E250A, N251A R**	CA$_2$T$_2$AT$_2$GCACTAGCAGCA$_2$TAT$_3$ACACTATCAT$_2$ACGACGATC$_2$ACAGC$_2$AT$_2$GA$_3$T$_2$C$_2$ATC
**SseK1 XhoI F**	CGCGCTCGAGATGATC$_3$AC$_2$AT$_2$A$_3$TAGATATGT$_2$C$_2$
**SseK1 KpnI F**	CGCG$_3$TAC$_3$TGCACATGC$_2$TCGC$_3$ATGA$_2$CT$_3$GCG
**SseK1 MseI F**	C$_2$AC$_2$ATGTAC$_3$ATACGATGT$_2$C$_2$AGAT$_2$ACGCTATGATC$_3$AC$_2$AT$_2$A$_3$TAG
**SseK1 BamHI R**	CTCATCA$_2$TGTATCT$_2$ATCATGTCTG$_2$ATC$_4$T$_2$ACTGCACATGC$_2$TCGC$_3$ATG
**SseK1 H244A F**	C$_2$G$_2$ATG$_2$A$_2$TCGCTGT$_2$GCTGTAGA$_3$G$_2$ATAGATG$_2$C$_2$
**SseK1 H244A R**	G$_2$C$_2$ATCTATC$_2$T$_3$CTACAGCA$_2$CAGCGAT$_2$C$_2$ATC$_2$G$_2$
**SseK1 E255A F**	GATAGATG$_2$C$_2$GTGCT$_2$CTATG$_2$CTA$_2$CG$_3$ATA$_2$TAGCTG
**SseK1 E255A R**	CAGCTAT$_2$ATC$_3$GT$_2$AGC$_2$ATAGA$_2$GCACG$_2$C$_2$ATCTATC
**SseK1 N265A F**	GATG$_2$C$_2$GTGCT$_2$CTATG$_2$A$_2$GC$_2$G$_3$ATA$_2$TAGCTGT$_2$GATCG
**SseK1 N256A R**	CGATCA$_2$CAGCTAT$_2$ATC$_3$G$_2$CT$_2$C$_2$ATAGA$_2$GCACG$_2$C$_2$ATC
**SseK1 H244A, E255A, N256A F**	G$_2$A$_2$TCGCTGT$_2$GCTGTAGA$_3$G$_2$ATAGATG$_2$C$_2$GTGCT$_2$CTATG$_2$CTGC$_2$G$_3$ATA$_2$TAGCTGT$_2$G
**SseK1 H244A, E255A, N256A R**	CA$_2$CAGCTAT$_2$ATC$_3$G$_2$CAGC$_2$ATAGA$_2$GCACG$_2$C$_2$ATCTATC$_2$T$_3$CTACAGCA$_2$CAGCGAT$_2$C$_2$
**SseK2 XhoI F**	CGCGCTCGAGATG$_2$CACGT$_4$A$_2$TGC$_2$GCT$_4$AC
**SseK2 KpnI R**	CGCG$_3$TAC$_4$TC$_2$A$_2$GA$_2$CTG$_2$CAGT$_2$A$_3$CTGCT$_2$G
**SseK2 H260A F**	C$_2$TGATG$_2$TATCGCTATCGCTGTA$_2$GTCGTA$_3$GATA$_2$TC
**SseK2 H260A R**	GAT$_2$ATCT$_3$ACGACT$_2$ACAGCGATAGCGATAC$_2$ATCAG$_2$
**SseK2 E271A F**	GATA$_2$TCATGT$_2$AGC$_2$T$_2$GCA$_3$TG$_3$AT$_2$AT$_2$GCTG
**SseK2 E271A R**	CAGCA$_2$TA$_2$TC$_3$AT$_3$GCA$_2$G$_2$CTA$_2$CATGAT$_2$ATC
**SseK2 N272A F**	GATA$_2$TCATGT$_2$AGC$_2$T$_2$GA$_2$GCTG3AT$_2$AT$_2$GCTGT$_2$A$_2$C

**SseK2 N272A R**	$GT_2A_2CAGCA_2TA_2TC_3AGCT_2CA_2G_2CTA_2CATGAT_2ATC$
**SseK2 H260A, E271A, N272A F**	$G_2TATCGCTATCGCTGTA_2GTCGTA_3GATA_2TCATGT_2AGC_2T_2GCAGCTG_3AT_2AT_2GCTG$
**SseK2 H260A, E271A, N272A R**	$CAGCA_2TA_2TC_3AGCTGCA_2G_2CTA_2CATGAT_2ATCT_3ACGACT_2ACAGCGATAGCGATAC_2$
**SseK2 D204A F**	$GACA_3G_2TCACT_2ATG_2T_3GC_2T_2CTACAGA_3TCT_4C$
**SseK2 D204A R**	$GA_4GAT_3CTGTAGA_2G_2CA_3C_2ATA_2GTGAC_2T_3GTC$
**SseK2 D239A F**	$G_2TG_3TGCATATATCT_2GCTGCAGATATGT_2ACT_2ACTG$
**SseK2 D239A R**	$CAGTA_2GTA_2CATATCTGCAGCA_2GATATATGCAC_3AC_2$
**TRADD BamHI F**	$TCGCG_2ATC_2ATG_2CAGCTG_3CA_4TG_3C$
**TRADD HindIII R**	$C_2GCA_2GCT_2G_2C_2AG_2C_2GC_2AT_2G_3ATC$

Supplementary Table 3. Plasmids used in this study

Description (Plasmids)	Source
**GST-NleB1**	Elqaidi et al 2017
**GST-NleB2**	Elqaidi et al 2017
**GST-SseK1**	Elqaidi et al 2017
**GST-SseK2**	Elqaidi et al 2017
**GST-NleB1 H242A**	This study
**GST-NleB1 E253A**	This study
**GST-NleB1 N254A**	This study
**GST-NleB1 H242A E253A N254A**	This study
**GST-NleB2 H239A**	This study
**GST-NleB2 E250A**	This study
**GST-NleB2 N251A**	This study
**GST-NleB2 H239A E250A N251A**	This study
**GST-SseK1 H244A**	This study
**GST-SseK1 E255A**	This study
**GST-SseK1 N256A**	This study
**GST-SseK1 H244A E255A N256A**	This study
**GST-SseK2 H260A**	This study
**GST-SseK2 E271A**	This study
**GST-SseK2 N272A**	This study
**GST-SseK2 H260A E271A N272A**	This study
**HA-NleB1**	This study
**HA-NleB1 H242A**	This study
**HA-NleB1 E253A**	This study
**HA-NleB1 N254A**	This study
**HA-NleB1 H242A E253A N254A**	This study
**HA-SseK1**	This study
**HA-SseK1 H244A**	This study
**HA-SseK1 E255A**	This study
**HA-SseK1 N256A**	This study
**HA-SseK1 H244A E255A N256A**	This study
**FLAG-NleB1**	This study
**FLAG-NleB1 H242A**	This study
**FLAG-NleB1 E253A**	This study
**FLAG-NleB1 N254A**	This study
**FLAG-NleB1 H244A E255A N256A**	This study
**FLAG-NleB2**	This study
**FLAG-NleB2 H239A**	This study

**FLAG-NleB2 E250A**	This study
**FLAG-NleB2 N251A**	This study
**FLAG-NleB2 H239A E250A N251A**	This study
**FLAG-SseK1**	This study
**FLAG-SseK1 H244A**	This study
**FLAG-SseK1 E255A**	This study
**FLAG-SseK1 N256A**	This study
**FLAG-Ssek1 H244A E255A N256A**	This study
**FLAG-SseK2**	This study
**FLAG-SseK2 H260A**	This study
**FLAG-SseK2 E271A**	This study
**FLAG-SseK2 N272A**	This study
**FLAG-SseK2 H260A E271A N272A**	This study
**FLAG-TRADD**	This study
**FLAG- TRADD DD**	This study
**FLAG-GAPDH**	This study
**His-TRADD**	This study
**His-GAPDH**	Elqaidi et al 2017
**His-FADD**	Elqaidi et al 2017
**GFP-TRADD**	This study
**GFP-FADD**	This study
**Myc-SseK1**	This study
**Myc-SseK1 AxA**	This study
**Myc-SseK1 H244A**	This study
**Myc-SseK1 E255A**	This study
**Myc-SseK1 N256A**	This study
**His-SseK1**	This study
**His-SseK1 21-336**	This study
**His-SseK1 H244A**	This study
**His-SseK1 E255A**	This study
**His-SseK1 N256A**	This study
**His-SseK1 W331A**	This study
**His-SseK1 F187A**	This study
**His-SseK1 AxA**	This study
**His-TRX-SseK2**	This study
**His-TRX-SseK2 34-348**	This study
**His-TRX-SseK2 H260A**	This study
**His-TRX-SseK2 E271A**	This study

**His-TRX-SseK2 N272A**	This study
**His-TRX-SseK2 F203A**	This study
**His-TRX-SseK2 W347A**	This study
**His-TRX-SseK2 AxA**	This study
**His-TRX-NleB2 (1-316)**	This study

Supplementary Table 4. Complete amino acid sequences used in this study

SseK1<br>(21-336, C39S, C210S)	MGSSHHHHHHSSGLVPRGSENLYFQSHMASMTGGQQMGRGSVTNRDIQFTSFNGKDYPL**S**FL<br>DEKTPLLFQWFERNPARFGKNDIPIINTEKNPYLNNIIKAATIEKERLIGIFVDGDFFPGQKDAFSKLE<br>YDYENIKVIYRNDIDFSMYDKKLSEIYMENISKQESMPEEKRDCHLLQLLKKELSDIQEGNDSLIKS<br>YLLDKGHGWFDFYRNMAMLKAGQLFLEADKVG**S**YDLSTNSGCIYLDADMIITEKLGGIYIPDGIAV<br>HVERIDGRASMENGIIAVDRNNHPALLAGLEIMHTKFDADPYSDGVCNGIRKHFNYSLNEDYNSFC<br>DFIEFKHDNIIMNTSQFTQSSWARHVQ*
SseK2<br>(34-348)	MGSSHHHHHHSSGLVPRGSHMSDKIIHLTDDSFDTDVLKADGAILVDFWAEWCGPCKMIAPILDEI<br>ADEYQGKLTVAKLNIDQNPGTAPKYGIRGIPTLLLFKNGEVAATKVGALSKGQLKEFLDANLASGTT<br>ENLYFQGSTLSPPSSGHVSFAGIDYPLLPLNHQTPLVFQWFERNPDRFGQNEIPIINTQKNPYLNNI<br>INAAIIEKERIIGIFVDGDFSKGQRKALGKLEQNYRNIKVIYNSDLNYSMYDKKLTTIYLENITKLEAQS<br>ASERDEVLLNGVKKSLEDVLKNNPEETLISSHNKDKGHLWFDFYRNLFLLKGSDAFLEAGKPGCH<br>HLQPGGGCIYLDADMLLTDKLGTLYLPDGIAIHVSRKDNHVSLENGIIAVNRSEHPALIKGLEIMHSK<br>PYGDPYNDWLSKGLRHYFDGSHIQDYDAFCDFIEFKHENIIMNTSSLTASSWR*
NleB2<br>(1-316, C21S, C199S)	MGSSHHHHHHSSGLVPRGSHMSDKIIHLTDDSFDTDVLKADGAILVDFWAEWCGPCKMIAPILDEI<br>ADEYQGKLTVAKLNIDQNPGTAPKYGIRGIPTLLLFKNGEVAATKVGALSKGQLKEFLDANLASGTT<br>ENLYFQGSMLSPIRTTFHNSVNIVQSSP**S**QTVSFAGKEYELKVIDEKTPILFQWFEPNPERYKKDE<br>VPIVNTKQHPYLDNVTNAARIESDRMIGIFVDGDFSVNQKTAFSKLERDFENVMIIYREDVDFSMY<br>DRKLSDIYHDIICEQRLRTEDKRDEYLLNLLEKELREISKAQDSLISMYAKKRNHAWFDFFRNLALL<br>KAGEIFR**S**TYNTKNHGISFGEGCIYLDMDMILTGKLGTIYAPDGISMHVDRRNDSVNIENSAIIVNRS<br>NHPALLEGLSFMHSKVDAHPYYDGLGKGVKKYFNFTPLHNYNHFCDFIEFNHPNIIM*

*Underline means vector-containing sequence and bold means point mutation site.

Supplementary Table 5. Chemical shift assignment of $GAPDH_{195-203}$(*)

Residue	GAPDH$_{195-203}$ Assignment Proton ID	^{1}H (ppm)	^{13}C (ppm)
Leu-195	HA	3.80	51.50
	HD	0.74	21.46
	HD	0.70	20.84
	HG	1.47	39.78
Trp-196	HA	4.49	55.01
	HB	3.06	26.73
	HD	7.04	124.17
	HE3	7.41	117.87
	HH2	6.93	119.06
	HZ2	7.28	111.61
	HZ3	7.02	121.70
Arg-197	HA	3.92	52.98
	HB	1.48	28.03
	HD	2.86	40.38
	HG	1.16	23.93
Asp-198	HA	4.16	51.23
	HB	2.48	37.75
Arg-200	HD	2.92	40.38
	HB	1.66	27.58
	HG	1.38	24.02
Gly-199/201	HA	3.75	42.63
	HA	3.64	42.42
Ala-202	HA	4.10	49.30
	HB	1.15	16.46
Leu-203	HA	3.97	53.63
	HB	1.37	40.24
	HB	1.42	23.81
	HD	0.69	22.19
	HD	0.64	20.57
	HG	0.71	20.59
	HA	3.80	51.50
	HD	0.74	21.46
	HD	0.70	20.84
	HG	1.47	39.78

(*) Due to strong overlapping some assignments are missing

Supplementary Table 6. Chemical shift assignment of $FADD_{110-118}$ (*)

Residue	$FADD_{110-118}$ Assignment Proton ID	^{1}H (ppm)	^{13}C (ppm)
Lys-110	HA	3.71	52.57
	HB	1.54	30.03
	HG	0.98	20.99
	HD	1.30	26.09
	HE	2.54	38.70
Asp-111	HA	4.52	50.59
	HB	2.57	37.62
Trp-112	HA	4.37	54.69
	HB	3.12	26.50
	HD1	7.02	121.65
	HE3	7.40	117.94
	HZ2	7.29	111.62
	HZ3	7.14	124.56
	HH2	6.94	119.01
Arg-113	HA	3.87	53.49
	HB	1.40	27.49
	HD	2.87	40.20
	HG	1.06	23.66
Arg-114	HA	3.98	53.27
	HB	1.43	24.03
	HD	2.99	40.20
	HG	1.36	24.21
Leu-115	HA	4.12	51.98
	HB	1.43	24.03
	HG	0.71	21.90
	HG	0.65	20.30
Ala-116	HA	4.09	49.75
	HB	1.19	16.20
Arg-117	HA	4.00	53.67
	HB	1.94	27.20
	HD	2.12	31.20
	HG	1.74	27.12
Gln-118	HA	4.10	53.26
	HB	1.59	27.61
	HG	2.99	40.20

(*) Due to strong overlapping some assignments are missing

Supplementary Table 7. Chemical shift assignment of TRADD$_{229\text{-}237}$ (*)

	TRADD$_{229\text{-}237}$ Assignment		
**Residue**	**Proton ID**	**^{1}H (ppm)**	**^{13}C (ppm)**
Lys-229	HA	3.78	52.76
	HB	1.67	-
	HD	1.48	-
	HE	2.77	39.09
	HG	1.21	-
Trp-230	HA	4.43	55.08
	HB	3.05	26.78
	HD	7.06	124.27
	HE3	7.38	117.91
	HH2	6.94	119.25
	HZ2	7.29	111.73
	HZ3	7.04	121.62
Arg-231	HA	3.95	52.45
	HB	1.40	-
	HD	2.89	40.46
	HG	1.26	-
Lys-232	HA	3.85	53.51
	HB	1.49	-
	HD	1.49	-
	HE	2.77	39.09
	HG	1.16	-
Val-233	HA	3.89	59.45
	HB	1.88	30.08
	HG	0.72	17.85
Gly-234	HA	3.77	42.02
Arg-235	HA	4.18	53.21
	HB	1.58	-
	HD	2.99	40.44
	HG	1.44	-
Ser-236	HA	4.24	55.52
	HB	3.66	60.93
Leu-237	HA	4.01	53.72
	HB	1.40	40.14
	HD1	0.70	22.22
	HD2	0.66	20.54
	HG	1.41	24.19

(*) Due to strong overlapping some assignments are missing

Supplementary References

1. Lamiable, A. et al. PEP-FOLD3: faster denovo structure prediction for linear peptides in solution and in complex. *Nucleic Acids Res.* **44**, W449-W454 (2016).
2. Kjaergaard, M. & Poulsen, F.M. Sequence correction of random coil chemical shifts: correlation between neighbor correction factors and changes in the Ramachandran distribution. *J. Biomol. NMR* **50**, 157-165 (2011).
3. Kjaergaard, M., Brander, S. & Poulsen, F.M. Random coil chemical shift for intrinsically disordered proteins: effects of temperature and pH. *J. Biomol. NMR* **49**, 139-149 (2011).
4. Wishart, D.S., Sykes, B.D. & Richards, F.M. The Chemical-Shift Index - a Fast and Simple Method for the Assignment of Protein Secondary Structure through Nmr-Spectroscopy. *Biochemistry* **31**, 1647-1651 (1992).
5. Wishart, D.S. & Sykes, B.D. The C-13 Chemical-Shift Index - a Simple Method for the Identification of Protein Secondary Structure Using C-13 Chemical-Shift Data. *J. Biomol. NMR* **4**, 171-180 (1994).
6. El Qaidi, S. et al. NleB/SseK effectors from Citrobacter rodentium, Escherichia coli, and Salmonella enterica display distinct differences in host substrate specificity. *J. Biol. Chem.* **292**, 11423-11430 (2017).
